# Antinociceptive potency of a fluorinated cyclopeptide Dmt-c[D-Lys-Phe-*p*-CF_3_-Phe-Asp]NH_2_

**DOI:** 10.1080/14756366.2018.1441839

**Published:** 2018-03-07

**Authors:** Justyna Piekielna-Ciesielska, Adriano Mollica, Stefano Pieretti, Jakub Fichna, Agata Szymaszkiewicz, Marta Zielińska, Radzisław Kordek, Anna Janecka

**Affiliations:** a Department of Biomolecular Chemistry, Faculty of Medicine, Medical University of Lodz, Lodz, Poland;; b Department of Pharmacy, University “‘G. d’Annunzio”’ of Chieti-Pescara, Chieti, Italy;; c Istituto Superiore di Sanità, National Center for Drug Research and Evaluation, Rome, Italy;; d Department of Biochemistry, Faculty of Medicine, Medical University of Lodz, Poland;; e Department of Pathology, Faculty of Medicine, Medical University of Lodz, Lodz, Poland

**Keywords:** Blood–brain barrier permeability, hot-plate test, anti-nociceptive and anti-inflammatory activity, colitis induction, myeloperoxidase activity

## Abstract

Opioid peptides and opiate drugs such as morphine, mediate their analgesic effects, but also undesired side effects, mostly through activation of the mu opioid receptor. However, delta- and kappa-opioid receptors can also contribute to the analgesic effects of opioids. Recent findings showed that simultaneous activation of multiple opioid receptors may result in additional analgesia with fewer side effects. Here, we evaluated the pharmacological profile of our formerly developed mixed mu/kappa-opioid receptor ligands, Dmt-c[D-Lys-Phe-Phe-Asp]NH_2_ (C-36) and Dmt-c[D-Lys-Phe-*p-*CF_3_-Phe-Asp]NH_2_ (F-81). The ability of these peptides to cross the blood–brain barrier was tested in the parallel artificial membrane permeability (PAMPA) assay. On the basis of the hot-plate test in mice after central and peripheral administration, analog F-81 was selected for the anti-nociceptive and anti-inflammatory activity assessment after peripheral administration.

## Introduction

Treatment of pain, especially chronic, is a common problem of all societies and one of the biggest challenges for modern medicine. Approximately 20% of adults suffer from the chronic pain syndrome (CPS)^1^. Opioids are the most effective drugs in chronic pain treatment but centrally mediated analgesia of opioids is often associated with some dangerous side effects such as respiratory depression, constipation, and also with tolerance and dependence^2^^,^^3^. The majority of clinically available opioid analgesics acting centrally are mu opioid receptor agonists derived mostly from morphine, with progressive simplification of its structure^4^.

The data collected so far suggest a strong contribution of peripheral mechanisms in pain suppression^5–7^. Opioid receptor ligands acting peripherally show less side effects as compared with those acting in the central nervous system (CNS)^5^^,^^8^^,^^9^. Peripheral mechanisms of pain control are especially effective in case of local inflammation processes when an increased number of opioid receptors on primary afferent neurons is observed^10–12^.

Endogenous opioid peptides were thought to be an alternative for morphine-based drugs, as they produced less side effects^13^^,^^14^. Unfortunately, their use as drugs is hampered by their short half-life in biological fluids. Additionally, degradation of these peptides increases in the microenvironment of the inflamed tissue, due to the elevated temperature, acidosis, and enhanced proteolytic activity of some enzymes^15–17^. Administration of more stable opioid peptide analogs produces significant peripherally-mediated analgesia in human^18–21^ and animal models^22–25^.

The anti-nociceptive and anti-inflammatory effects are mediated mostly by activation of the peripheral mu- and kappa-opioid receptors^26^. Thus, the use of multifunctional opioid analogs, which simultaneously activate more than one receptor, may generate a better drug profile, with improved potency and/or reduced side effects^27^. Therefore, extensive structure–activity relationship studies of opioid peptides are focused on the development of analogs with various activity profiles and enhanced efficacy^28–30^.

Here, we characterized the artificial membrane permeability and anti-nociceptive activity in the mouse hot-plate test of our two formerly developed analogs Dmt-c[D-Lys-Phe-Phe-Asp]NH_2_ (C-36)^31^ and Dmt-c[D-Lys-Phe-*p-*CF_3_-Phe-Asp]NH_2_ (F-81)^32^, with the mu/kappa-opioid receptor profile. On the bases of the obtained results, F-81 was chosen for further evaluation as a peripherally restricted peptide with possible anti-nociceptive and anti-inflammatory activity.

## Materials and methods

### Kinetic solubility

Kinetic solubility assay was performed using Multiscreen HTS Vacuum Manifold (Merck Millipore, Warsaw, Poland). Compounds for this test were prepared as 10 mM stock solutions in DMSO and diluted in a standard PBS buffer (pH = 7.4), to the final concentrations of 0.5 mM. The solutions were applied into a 96-well filter plate and incubated for 90 min at room temperature, while shaking at 40 g and then filtrated. The concentration of each compound was determined on the basis of the prepared calibration curve using the UV–VIS spectrophotometric method. According to the absorption spectra of the compounds the wavelength used for the measurements was 280 nm for both, C-36 and F-81.

### Parallel Artificial Membrane Permeability Assay

Parallel Artificial Membrane Permeability Assay (PAMPA) was performed using Multiscreen Filter Plate and Multiscreen Transport Receiver Plate (Merck Millipore, Warsaw, Poland). Tested, as well as reference compounds, were dissolved in 0.1 M PBS buffer containing 20% of ethanol, to the final concentration of 200 µM. A 300 µl aliquots of 0.1 M PBS solution (pH = 7.4) was dispensed into the wells of the donor chamber. Then 1% *n*-dodecane solution of the brain polar lipid extract (Avanti Polar Lipids; Sigma Aldrich, Poznan, Poland) was prepared to investigate the blood–brain barrier (BBB) permeability. Aliquots of the obtained lipid solutions (5 µl) were dispensed on the membranes of wells of the donor plate and left to dry. Then, solutions of the tested and reference (verapamil and theophylline) compounds (150 µl) were added into the wells of the donor plate. A complete system, consisting of the receiver plate with inserted donor plate, was incubated at room temperature for 4 h, shaking at 40 g, to guarantee good solvent mixing. Then, the samples were collected, concentration of the tested compounds in both compartments was determined using UV–VIS spectrophotometer (wavelength 280 nm for verapamil, C-36 and F-81, and 270 nm for theophylline, respectively) and the permeability coefficients (*P*_e_) were calculated.

The ability of the tested compounds to permeate the artificial membranes was classified according to the literature^33^ as:

*P*_e_ > 4.0 – high permeation,

*P*_e_ < 2.0 – low permeation,

*P*_e_ from 2.0 to 4.0 – permeation uncertain.

### Animals

Mice were housed for at least 1 week before the experimental sessions in sawdust-lined plastic cages (7–10 mice in each cage). Animals were housed at a constant temperature (21 ± 1 °C) and maintained under a 12 h light/dark cycle with food and water available *ad libitum*.

Male CD-1 mice (Harlan, Italy) weighing 25–30 g were used to determine the anti-nociceptive activity of the cyclopeptides in the hot-plate test after i.c.v. injection. Animal studies and research protocols were approved by the Service for Biotechnology and Animal Welfare of the Istituto Superiore di Sanità and authorized by the Italian Ministry of Health, according to Legislative Decree 26/14 (protocol number: 198/2013-B, 14 August 2013), which implemented the European Directive 2010/63/EEC on laboratory animal protection. Animal welfare was routinely checked by veterinarians from the Service for Biotechnology and Animal Welfare.

Male balb/c mice (Animal Facility of Nofer Institute of Occupational Medicine, Lodz, Poland) weighing 22–25 g were used to determine the anti-inflammatory and anti-nociceptive activity of cyclopeptides after i.p. administration. All procedures and protocols used in this study were approved by the Local Ethical Committee for Animal Research (protocol number: 20/ŁB708/2014, 26 May 2014). The study was carried out in accordance with the recommendations described in the Guide for the Care and Use of Laboratory Animals of the Medical University of Lodz, Poland. All experimental procedures were performed in accordance with the current guidelines for the care of laboratory animals (including the use of the 3Rs procedures) and in accordance with the ARRIVE guidelines^34^.

### Assessment of anti-nociception after i.c.v. administration (hot-plate test)

The hot-plate test was performed as described earlier^35^. The intracerebroventricular (i.c.v.) injections were performed in a volume of 10 µl/animal, in the left brain ventricle of manually immobilized mice with a Hamilton microsyringe (50 µl) connected to a needle (diameter 0.5 mm). Peptides were also administrated in mice intravenously (i.v.) in a volume of 1 ml/kg. All compounds used for i.c.v. and i.v. administration were dissolved in saline. A transparent plastic cylinder (14 cm diameter, 31 cm height) was used to confine the mouse on the heated (55 ± 0.5 °C) surface of the plate. The animals were placed on the hot plate 5 min after the i.c.v. injection of saline (control) or peptides and the latencies to paw licking, rearing and jumping were measured at 5, 10, 20, 30, 45, 60, 90, and 120 min after administration of a peptide. A cut-off time of 240 s was used to avoid tissue injury. The percentage of the maximal possible effect (%MPE) was calculated as: %MPE = (*t*_1_ − t_0_)/(*t*_2_ − *t*_0_) × 100, where *t*_0_: control latency, *t*_1_: test latency, and *t*_2_: cut-off time. The median antinociceptive dose (ED_50_) was calculated according to the method of Litchfield and Wilcox^36^.

### Induction of colitis and assessment of colonic damage

The experimental colitis was induced by intracolonic (i.c.) administration of 2,4,6-trinitrobenzenesulfonic acid (TNBS). Mice were anesthetized with isoflurane (Aerrane, Baxter, Deerfield, IL) and TNBS at the dose of 4 mg per mouse (dissolved in 30% EtOH in saline) in the final volume of 150 µl, was injected into the colon through a catheter inserted 3 cm proximally from the anus. Mice were then maintained in an inclined position for 1 min to ensure the proper distribution of the inductor in the colon. Next, recovery was allowed with food and water supplied. Control animals received vehicle alone (30% ethanol in saline; TNBS replaced with equivolume water).

The preventive effect of F-81 on the colitis development as well as its anti-inflammatory properties in the semi-chronic colitis model were characterized, allowing to determine therapeutic activity of the tested compound^37^.

In the prevention mouse model of colitis, F-81 was injected intraperitoneally (i.p.) at the dose of 1 mg/kg in saline, in the volume of 100 µl, once daily, for 3 days, 15 min prior to TNBS administration. Mice were weighted daily and monitored for clinical symptoms of colitis, including diarrhoea and bloody stool. On day 4, mice were sacrificed by rapid cervical dislocation and the macroscopic damage score was assessed.

In the semi-chronic model, the curative potential of F-81 was tested. TNBS was administered on day 0 and from day 3 to day 6 the F-81 treatment (1 mg/kg in saline, 100 µl, i.p) was performed. On day 7, mice were sacrificed and the evaluation of colonic damage was performed.

To perform the macroscopic damage score, the colon was removed immediately after euthanasia, opened longitudinally, rinsed with phosphate buffered saline (PBS), and examined using an established semiquantitative scoring system by adding individual scores for ulcer, colonic shortening, wall thickness, and the presence of haemorrhage, faecal blood, and diarrhoea, as described before^37^. For scoring ulcer and colonic shortening the following scale was used: ulcer: 0.5 points for each 0.5 cm; shortening of the colon: 1 point for >15%, 2 points for >25% (based on a mean length of the colon in untreated mice). The wall thickness was measured in millimetres, a thickness of *n* mm corresponded to *n* scoring points. The presence of haemorrhage, faecal blood, or diarrhoea increased the score by 1 point for each additional feature.

### Histology

Distal colon sections from the preventive mouse model of colitis were stapled flat, mucosal side-up, onto cardboard strips and fixed in 10% formalin for 24 h at 4 °C. Samples were dehydrated, embedded in paraffin, sectioned at 5 µm, and mounted onto slides. The sections were then stained with haematoxylin and eosin and Motic AE31 microscope (Ted Pella, Sweden) photographs were taken using a digital imaging system consisting of a digital camera (Moticam 2300, Ted Pella, Sweden) and image analysis software (Motic Images Plus 2.0, Germany).

The microscopic total damage score was assessed using the following parameters: the goblet cell depletion (presence = 1, absence = 0), crypt abscesses (presence = 1, absence = 0), the destruction of mucosal architecture (normal = 1, moderate = 2, extensive = 3), the extent of muscle thickening (normal =1, moderate = 2, extensive = 3), and the presence and degree of cellular infiltration (normal = 1, moderate = 2, transmural = 3).

### Determination of tissue myeloperoxidase (MPO) activity

The colon fragments (20–30 mg) were gently isolated from mice after total macroscopic damage score assessment, immediately washed with PBS, and homogenized in hexadecyltrimethylammonium bromide (HTAB) buffer (0.5% HTAB in 50 mM potassium phosphate buffer, pH 6.0; 50 mg of tissue/ml) using Ika Ultra Turrax Disperser T25 Digital 2 (Sigma Aldrich, Poznan, Poland). The homogenates were centrifuged (20 min, 13,200*g*, 4 °C) and the supernatants were transferred to the new test tubes. Then, 7 µl portions of supernatants were added on a 96-well plate, followed by 200 µl of 50 mM potassium phosphate buffer (pH = 6.0), containing 0.167 mg/ml of O-dianisidine hydrochloride and 0.05 µl of 1% H_2_O_2_. The absorbance was measured at 450 nm after 30 and 60 s (iMARK Microplate Reader, Biorad, UK). All measurements were done in triplicates.

The MPO activity was expressed in units/g of a wet tissue. One unit is determined as a quantity of enzyme able to convert 1 µmol of H_2_O_2_ to water in 1 min at room temperature. MPO activity units were calculated from the standard curve using purified peroxidase enzyme.

### Mustard oil induced pain

To assess the anti-nociceptive effect of F-81 in mice with acute colitis, intestinal inflammation was induced by TNBS instillation three days prior to the experiment. Abdominal pain was induced in inflamed mice by the i.c. injection of 1% mustard oil (MO, allyl isothiocyanate) in 70% EtOH in saline. Mice were separated into clear plastic boxes (20 × 20 × 15 cm) and allowed a 5 min recovery after MO administration. Spontaneous behaviours (licking and stretching the abdomen, squashing of lower abdomen against the floor, and abdominal retraction) were observed and counted for 20 min.

### Data analysis and terminology

Artificial membrane permeability was assessed in the PAMPA assay and the permeability coefficient (Pe) was calculated from the following equation:

$$Pe=C\times -ln\left(1-\times \frac{C_{a}}{C_{r}}\right)$$

where

$$C=\frac{V_{d\;\;}{\times V}_{a\;}}{(V_{d}+V_{a})\times p\times t}$$

Membrane retention was analysed using mass balance between a compound left in the donor and acceptor compartments and calculated using the following equation:

$$MB=\frac{\left(C_{a}\times V_{a}\right)+(C_{d}\times V_{d})}{C_{initial}\times V_{d}}$$

where

*V*_a_ – volume of acceptor compartment,

*C*_a_ – concentration in acceptor compartment (after incubation),

*V*_d_ – volume of donor compartment,

*C*_d_ – concentration in donor compartment (after incubation),

*C*_initial_ – initial concentration in donor compartment,

*C*_r_ – concentration in equilibrium,

*p* – membrane surface area (0.28 cm^2^),

*t* – time (s).

## Results

### Kinetic solubility

Kinetic solubility allows for determination of a compound solubility, based on the precipitation process. It is widely used at the early stages of drug discovery to assess the solubility of compounds in *in vitro* conditions in pharmacological assays.

In 0.1 M PBS (pH 7.4), C-36 and F-81 were both highly soluble (500 and 345 µM, respectively) (Table 1). The solubility of the tested peptides was sufficient to perform the PAMPA assay.

**Table 1.** The kinetic solubility test results.

		Solubility		Solubility	
Peptide	MW (g/mol)	Mean (µM)	SD	Mean (mg/ml)	SD
C-36	727	≥500	–	≥0.36	–
F-81	795	345	15	0.27	0.01

### Membrane permeability assay

PAMPA was used to assess passive permeability of cyclopeptides across the artificial BBB. Both tested compounds showed low permeation across this barrier (Table 2).

**Table 2.** Blood–brain barrier permeability (PAMPA) results for cyclic pentapeptide analogs.

Compound	BBB permeability *P*_e_ (10^−6^ cm^−1^) ± SD	Mean (%) Mass balance ± SD	Classification
Verapamil	29.9 ± 6.5	89.28 ± 2.35	High permeation
Theophylline	≤0.58	98.21 ± 3.65	Low permeation
C-36	≤0.58	97.87 ± 1.69	Low permeation
F-81	≤0.58	95.75 ± 1.43	Low permeation

### Assessment of anti-nociceptive activity

**Figure 1.**
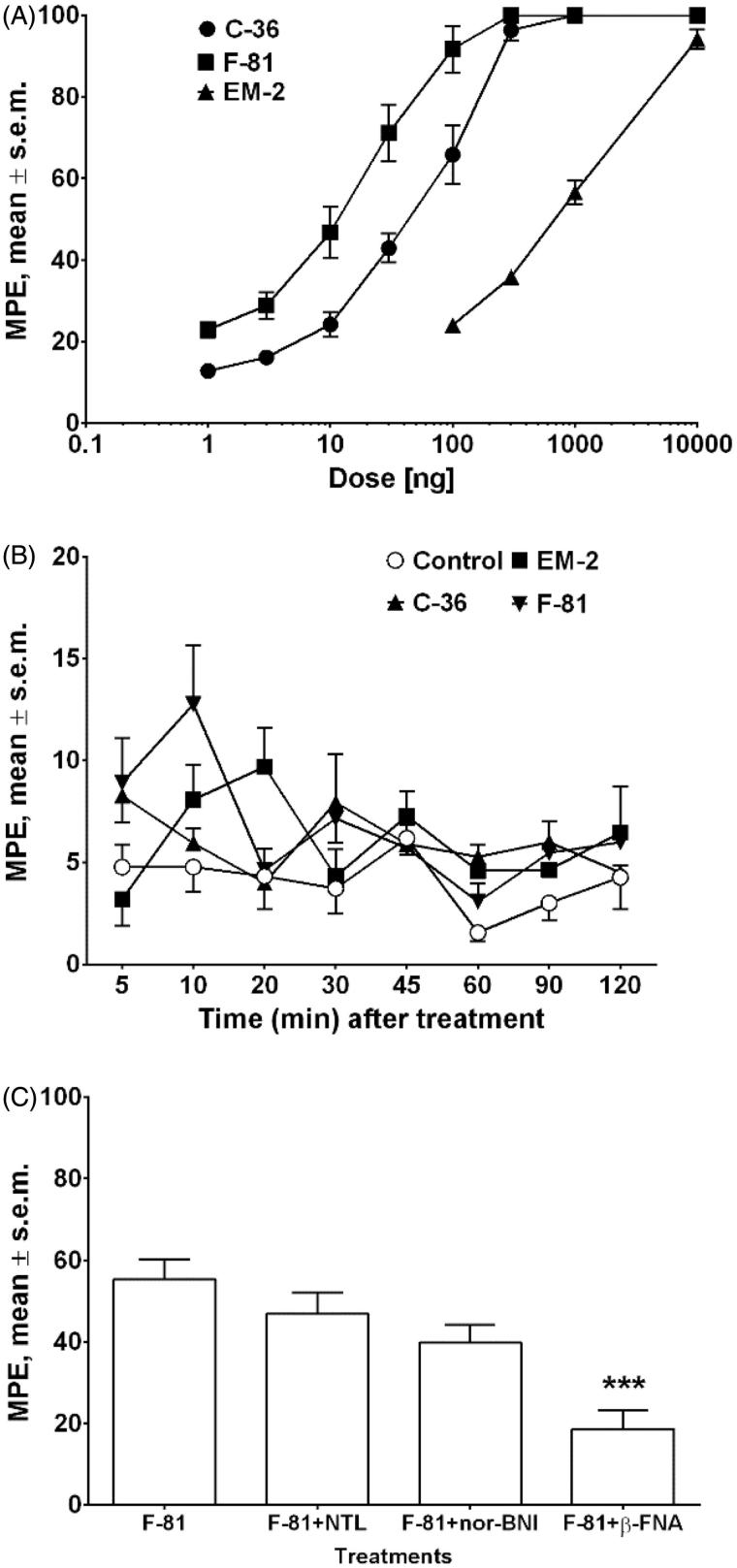
Dose–response curves determined in the hot-plate test for the inhibition of jumping induced by i.c.v. injection of EM-2 and cyclic analogs C-36 and F-81 (Panel A). Effects induced by peptides after i.v. administration at the dose of 20 mg/kg (Panel B). The antagonist effect of β-funaltrexamine (β-FNA, 1 µg/animal, i.c.v.), naltrindole (NTL, 1 µg/animal, i.c.v.), and nor-binaltorphimine (nor-BNI, 5 µg/animal, i.c.v.), on the inhibition of jumping induced by F-81 (10 ng/animal, i.c.v.) (Panel C). *** is for *p* <.001 for agonist + β-FNA versus agonist alone using two-way ANOVA, followed by the Student–Newman– Keuls test. The data represent mean ± SEM of 10 mice per group.

Anti-nociception was studied in the hot-plate test in mice after i.c.v. or i.v. administration of peptides. The results obtained in the dose-response studies after i.c.v. administration are shown in Figure 1(A). Both tested compounds showed dose-dependent anti-nociceptive activity, significantly stronger than that of endomorphin-2 (EM-2). The ED_50_ values (jumping response) for C-36 and F-81 were 57.78 and 17.27 ng, respectively, indicating that F-81 was approximately threefold more potent than C-36 (Figure 1(A)). In order to investigate if these peptides are able to cross the BBB, peripheral i.v. administration of the peptides was performed, and the results are reported in Figure 1(B). After i.v. administration at the dose of 20 mg/kg, only a negligible anti-nociceptive activity was observed for both compounds (Figure 1(B)). To characterize the involvement of opioid receptors in the anti-nociceptive action of analog F-81, co-administration studies with opioid receptor antagonists were performed. The anti-nociceptive effect of F-81 (10 ng/animal, i.c.v.) was blocked by β-funaltrexamine (β-FNA, 1 µg/animal), showing the involvement of the mu opioid receptors. The delta-opioid receptor antagonist, naltrindole (NTL, 1 µg/animal), and kappa-opioid receptor antagonist, norbinaltorphimine (nor-BNI, 5 µg/animal, i.c.v.), did not modify the anti-nociceptive action of F-81 (Figure 1(C)). Even though F-81 and C-36 showed significant kappa-affinity, the obtained results are in agreement with a generally accepted fact that the anti-nociceptive effects are mainly mediated by the mu opioid receptor^32^^,^^38^.

### Assessment of anti-inflammatory and anti-nociceptive activity of F-81 after peripheral administration

**Figure 2.**
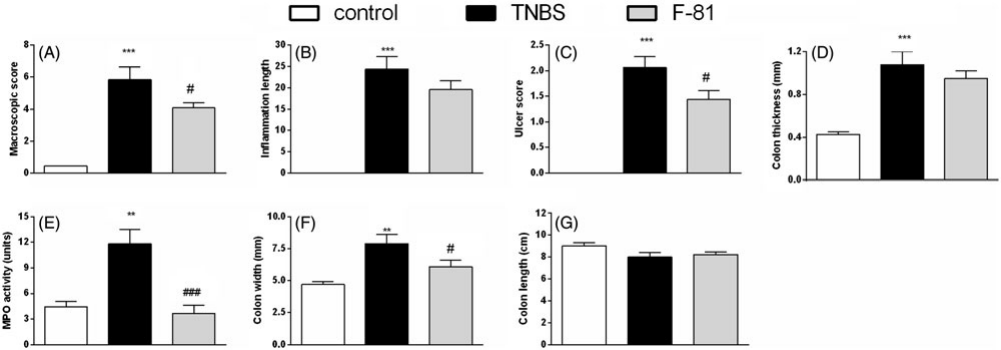
Anti-inflammatory activity of F-81, administered i.p. once a day over 3 days, in the prevention mouse model of the TNBS-induced colitis. Macroscopic score (Panel A), inflammation area (Panel B), ulcer score (Panel C), colon thickness (Panel D), MPO activity (Panel E), colon width (Panel F), and colon length (Panel G). Statistical significance was assessed using one-way ANOVA and *post hoc* multiple comparison Student–Newman–Keuls test. ***p < .001, as compared with controls. ^#^*p* < .05, ^###^*p* < .001, vs. TNBS-treated animals. Data represent mean ± SEM of eight mice per group.

**Figure 3.**
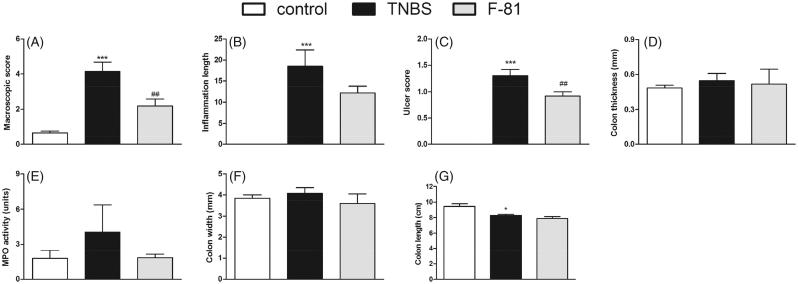
Anti-inflammatory activity of F-81, injected i.p. ameliorated experimental colitis in the semi-chronic TNBS-induced model. Macroscopic score (Panel A), inflammation area (Panel B), ulcer score (Panel C), colon thickness (Panel D) MPO activity (Panel E), colon width (Panel F), and colon length (Panel G). Statistical significance was assessed using one-way ANOVA and *post hoc* multiple comparison Student–Newman–Keuls test. ***p < .001, as compared with controls. ^#^*p* < .05, ^###^*p* < .001, vs. TNBS-treated animals. Data represent mean ± SEM of eight mice per group.

The i.c. administration of TNBS induced colonic inflammation in mice, as shown by the increased macroscopic damage score, ulceration of the intestinal wall and inflammation area which were significantly enhanced in the TNBS-treated group, as compared with the control group. The colon thickness and width were increased and colon length was decreased in the TNBS treated mice. Additionally, the MPO activity, which is an indicator of immune cell infiltration, was significantly enhanced in inflamed mice (Figures 2 and 3).

In the prevention model of TNBS-induced colitis, F-81 (injected i.p. at the dose of 1 mg/kg, once daily) decreased macroscopic damage score (4.07 vs. 5.83 for F-81 and TNBS-treated group, respectively), the inflammation area (19.56 ± 2.16 vs. 24.42 ± 2.92), improved the ulcer score (1.43 ± 0.17 vs. 2.06 ± 0.22) and improved the colon width (6.09 ± 0.53 vs. 7.91 ± 0.23) as compared with the TNBS-treated animals. There were no differences in the intestinal wall thickness (0.95 ± 0.07 vs. 1.08 ± 0.12) and the colon length 8.23 ± 0.24 vs. 8.01 ± 0.39 (in comparison with inflamed mice). The activity of MPO was significantly decreased in F-81-treated mice as compared with the TNBS-treated group (3.65 ± 0.87 vs. 11.82 ± 1.72, respectively) (Figure 2(A–G)).

In the semi-chronic model of TNBS-induced colitis (Figure 3(A–G)), the anti-inflammatory effect of F-81 was shown by the decrease in the macroscopic damage score (2.18 ± 0.40 vs. 4.15 ± 0.54 for F-81- and TNBS-treated group, respectively) and inflammation area (12.23 ± 1.56 vs. 18.49 ± 3.95) and by improved ulcer score (0.92 ± 0.83 vs. 1.30 ± 0.12). No differences were observed in the colon thickness, width, or length between the F-81-treated and TNBS-treated groups. The MPO activity was also decreased in this model in the F-81-treated mice (7.88 ± 0.24 vs. 8.26 ± 0.15) (Figure 3(E)).

**Figure 4.**
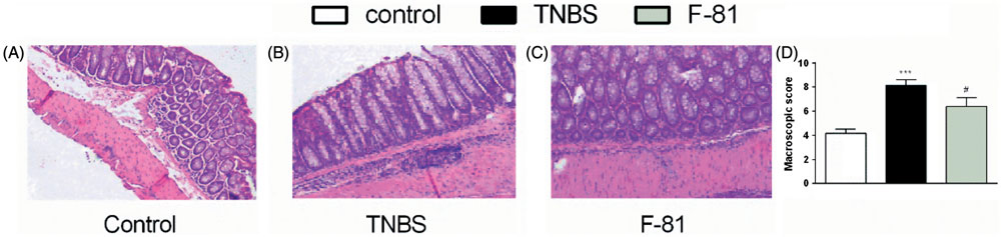
Representative images of haematoxylin- and eosin-stained sections of distal colon obtained from the prevention mouse model of colitis for control (Panel A), TNBS-treated group (Panel B), TNBS- and F-81-treated group (Panel C). Microscopic damage score (Panel D**)**. Statistical significance was assessed using one-way ANOVA and *post hoc* multiple comparison Student–Newman–Keuls test. ***p < .001, as compared with controls. ^#^*p* < .05, ^###^*p* < .001, vs. TNBS-treated animals. Data represent mean ± SEM of eight mice per group.

Microscopically, the induction of inflammation by TNBS was associated with a destruction of the intestinal wall (including architecture of mucosa and the presence of goblet cells and crypts), infiltration of immune cells and thickening of the muscle layer, as compared with naive animals (Figure 4(A–D)). The administration of F-81 (1 mg/kg) significantly improved the microscopic score (Figure 4(D)).

**Figure 5.**
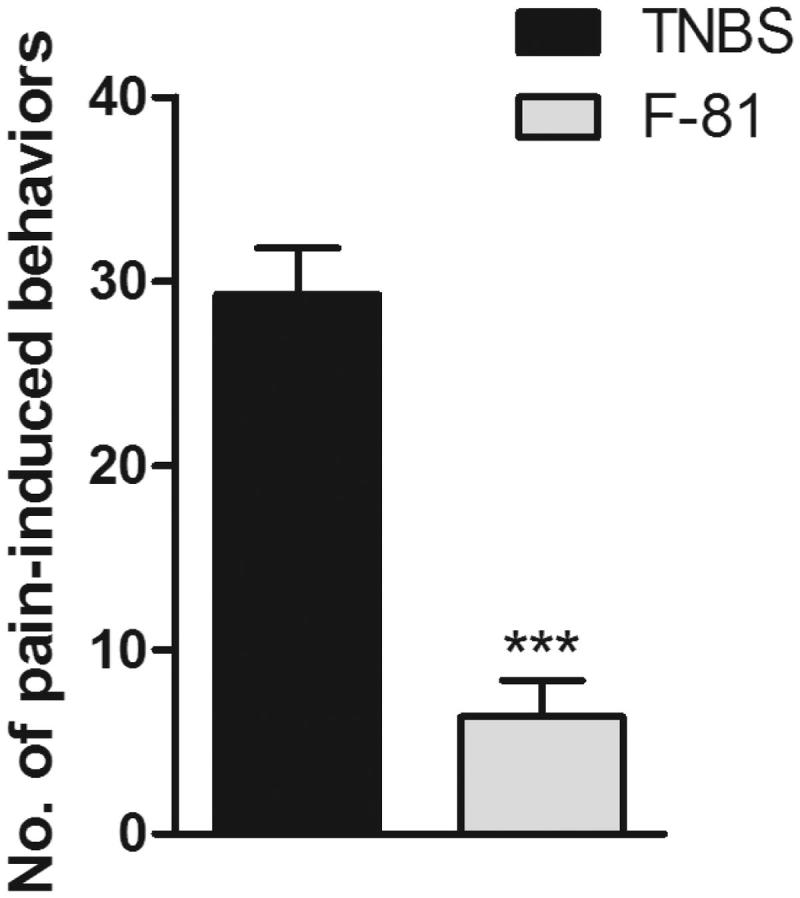
The anti-nociceptive effect of F-81 in the mustard oil-induced pain in mice with acute TNBS-induced colitis. Data represent mean ± SEM of eight mice per group. Statistical significance was assessed using *t*-test. ***p < .001, as compared with the control group.

The anti-nociceptive effect of F-81 was also characterized in the mouse model of abdominal pain induced by mustard oil instillation. Mice with TNBS-induced colitis were injected with F-81 (1 mg/kg, i.p.). This cyclopeptide significantly decreased the number of spontaneous pain behaviours; a strong anti-nociceptive activity was observed as compared with the control group (6.36 ± 1.98 vs. 29.28 ± 2.52, respectively) (Figure 5).

## Discussion

Opioid receptors are localized in the CNS and also in many peripheral tissues. The anti-nociceptive effects of opioid ligands given peripherally depends on their ability to cross the BBB. Ligands that can penetrate this barrier produce both, central and peripheral effects, while the action of those unable to reach the CNS is limited to the peripheral tissues. Peripherally restricted opioid analgesics, devoid of centrally mediated side effects can be a safer alternative for treating inflammatory painful disorders of gastrointestinal tract, skin, and joints^39^^,^^40^.

Endogenous opioid peptides elicit strong analgesic effect, however their usefulness after peripheral administration is very limited due to the rapid degradation in biological fluids^41^.

For several years, we have been engaged in the development of new, more stable opioid analogs with improved pharmacological profile. In this study, we tested the ability of cyclopeptides C-36 and F-81 (both with Dmt^1^) to cross the artificial BBB, using PAMPA. The PAMPA assay was previously shown to closely match the BBB membrane in such characteristics as hydrophobicity, rigidity, and fluidity, and is considered as an accurate passive diffusion permeability model for BBB^42^.

In the PAMPA experiment, both compounds showed low permeation through this artificial barrier. These results were confirmed *in vivo* in the mouse hot-plate test, since no anti-nociceptive activity was observed after peripheral (i.v.) administration of the tested compounds.

C-36 differs from our formerly published analog Tyr-c[D-Lys-Phe-Phe-Asp]NH_2_ [35] only by the presence of Dmt^1^ instead of Tyr^1^. As opposed to C-36, Tyr^1^-containing analog could reach the brain after i.v. administration. Recently, Zadina et al.^30^ reported several cyclopeptides of a similar structure also containing Tyr^1^ and those analogs were able to produce strong analgesia after peripheral administration. Therefore, the presence of Dmt^1^ in cyclic peptides may be a structural element responsible for lower permeability of such analogs through biological barriers.

However, the observed effect can be caused by other factors. Weltrowska et al.^43^ studied brain uptake of linear and cyclic DALDA (Tyr-D-Arg-Phe-Lys-NH_2_) analogs, both containing Dmt^1^. They observed a lower brain uptake and faster efflux of the more rigid cyclic peptide, indicating that structural flexibility strongly influences BBB permeability.

Since peripheral opioid receptors are involved in analgesia, especially in the presence of inflammation process, we decided to test the anti-nociceptive and anti-inflammatory activity of one of the analogs in abdominal pain models after peripheral administration. For these experiments F-81 was chosen, as its anti-nociceptive activity was threefold higher than that of C-36 in the mouse hot-plate test after central administration. The obtained data indicated that F-81 significantly attenuated inflammation and evoked strong anti-nociception in the mouse models of TNBS-induced colitis.

The observed effect of F-81 in the gastrointestinal tract is an interesting finding and can be useful in the further development of peripherally restricted agents.
